# The Proteomic Signature of Intestinal Acute Rejection in the Mouse

**DOI:** 10.3390/metabo12010023

**Published:** 2021-12-27

**Authors:** Mihai Oltean, Jasmine Bagge, George Dindelegan, Diarmuid Kenny, Antonio Molinaro, Mats Hellström, Ola Nilsson, Carina Sihlbom, Anna Casselbrant, Marcela Davila, Michael Olausson

**Affiliations:** 1 The Transplant Institute, Sahlgrenska University Hospital, 413 45 Gothenburg, Sweden; michael.olausson@transplant.gu.se; 2 Laboratory for Transplantation and Regenerative Medicine, Institute of Clinical Sciences, Sahlgrenska Academy at the University of Gothenburg, Sahlgrenska Science Park Medicinaregatan 8, 413 90 Gothenburg, Sweden; jasmine.bagge@gu.se (J.B.); mats.hellstrom@gu.se (M.H.); 3 First Surgical Clinic, Str. Clinicilor 3-5, 400006 Cluj-Napoca, Romania; george.dindelegan@umfcluj.ro; 4 Faculty of Medicine, University of Medicine and Pharmacy Cluj-Napoca, 400000 Cluj-Napoca, Romania; 5 Proteomics Core Facility, Sahlgrenska Academy, University of Gothenburg, Medicinaregatan 5, 413 90 Gothenburg, Sweden; diarmuidkenny@gmail.com (D.K.); carina.sihlbom@gu.se (C.S.); 6 Wallenberg Laboratory, Department of Molecular and Clinical Medicine, Sahlgrenska Academy, University of Gothenburg, 413 45 Gothenburg, Sweden; antonio.molinaro@wlab.gu.se; 7 Sahlgrenska Center for Cancer Research, Department of Laboratory Medicine, Institute of Biomedicine, Sahlgrenska Academy, University of Gothenburg, 405 30 Gothenburg, Sweden; ola.nilsson@llcr.med.gu.se; 8 Department of Surgery, Institute of Clinical Sciences, Sahlgrenska Academy, the University of Gothenburg, 413 45 Gothenburg, Sweden; anna.casselbrant@gastro.gu.se; 9 Bioinformatics Core Facility, University of Gothenburg, Medicinaregatan 5, 413 90 Gothenburg, Sweden; marcela.davila@gu.se

**Keywords:** intestinal transplantation, rejection, biomarkers, enzymes, chromogranin A

## Abstract

Intestinal acute rejection (AR) lacks a reliable non-invasive biomarker and AR surveillance is conducted through frequent endoscopic biopsies. Although citrulline and calprotectin have been suggested as AR biomarkers, these have limited clinical value. Using a mouse model of intestinal transplantation (ITx), we performed a proteome-wide analysis and investigated rejection-related proteome changes that may eventually be used as biomarkers. ITx was performed in allogenic (Balb/C to C57Bl) and syngeneic (C57Bl) combinations. Graft samples were obtained three and six days after transplantation (n = 4/time point) and quantitative proteomic analysis with iTRAQ-labeling and mass spectrometry of whole tissue homogenates was performed. Histology showed moderate AR in all allografts post-transplantation at day six. Nine hundred and thirty-eight proteins with at least three unique peptides were identified in the intestinal grafts. Eighty-six proteins varying by >20% between time points and/or groups had an alteration pattern unique to the rejecting allografts: thirty-seven proteins and enzymes (including S100-A8 and IDO-1) were significantly upregulated whereas forty-nine (among other chromogranin, ornithine aminotransferase, and arginase) were downregulated. Numerous proteins showed altered expression during intestinal AR, several of which were previously identified to be involved in acute rejection, although our results also identified previously unreported proteome changes. The metabolites and downstream metabolic pathways of some of these proteins and enzymes may become potential biomarkers for intestinal AR.

## 1. Introduction

The enterocytes abundantly express class II major histocompatibility complex molecules, making the intestinal lining highly immunogenic and susceptible to acute rejection (AR). During AR, recipient T-cells initially leave the intravascular compartment and infiltrate the lamina propria of the graft and ultimately attack graft enterocytes, eventually leading to mucosal loss. Unlike kidney and liver grafts, the acute rejection (AR) of the intestinal allograft lacks reliable non-invasive markers. The need for a biomarker of intestinal AR is pressing as AR may lead to graft and patient loss [1,2]. Given this lack of biomarkers, the current strategy for rejection surveillance still relies heavily on frequent protocol endoscopies and mucosal biopsies, which incur logistic issues, risks, and costs [1,3]. Depending on its stage, the histology of intestinal AR reveals various degrees of inflammation in the lamina propria, increased crypt apoptosis, crypt damage, crypt loss, villous blunting with edema, and congestion, culminating in mucosal sloughing. Alterations in the metabolism of several biomolecules such as decrease in plasma citrulline or increased fecal calprotectin have been reported during intestinal AR, and these two parameters were suggested as non-invasive rejection biomarkers. Unfortunately, both are influenced by numerous factors including the renal function, body surface area, infectious enteritis, or reperfusion injury. Hence, the significant inter-individual variation and their low specificity (50–75%) make them unreliable [4,5] and a search for other reliable biomarkers, suitable for safe, routine measurements is warranted.

Changes in the cellular expression of various molecules have been reported during intestinal AR in both the experimental and clinical setting. These changes appear secondary to different biological processes such as inflammation [6,7,8], tissue injury and repair [9,10], cell metabolism or apoptosis [11]. Two studies using proteomics and metabolomics to analyze the stomal effluent in intestinal transplant patients revealed complex patterns in the metabolism of various molecules released into the intestinal lumen, many of which seemed related to the AR. One study showed a clear interclass separation of metabolites detected during episodes of rejection with several metabolites related to leukotriene E4 and water soluble vitamins [12]. Another study identified 17 distinct protein expression profiles altered during rejection including human neutrophil peptide (HNP) 1, HNP 2, and human α-defensin 5 [13]. Despite the interesting and comprehensive data, and the undeniable clinical relevance, clinical studies have several limitations such as concurrent medication, the contribution of other digestive organs, or patient and sampling heterogeneicity [14,15].

Experimental transplantation in mice offers unique advantages such as highly standardized experimental conditions, a similar surgical procedure, and the advantage of a fully mapped proteome. Moreover, the investigator may easily influence the timing of rejection by the use of various strain combinations [16], select endpoints, and analyze rejection-related changes without the involvement of immunosuppressants. In the current study, we performed a proteome-wide analysis of the protein changes that occurred within the intestinal graft prior to, and during the acute rejection using isobaric tags for relative and absolute quantitation (iTRAQ). We then analyzed and compared protein expression changes in rejecting grafts (allogeneic combination) with non-rejecting (syngeneic) grafts.

## 2. Results

### 2.1. Intestinal Graft Histology

**Figure 1:**
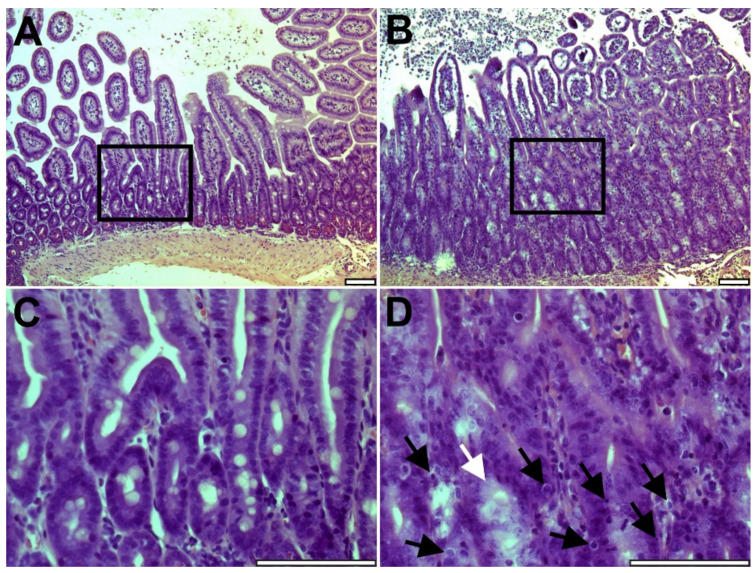
Histology of the intestinal allografts three days (**A**,**C**) and six days after transplantation (**B**,**D**) at low (100×) and high (400×) magnification showing essentially normal histology after three days and moderate acute rejection six days after transplantation with swollen villi, lymphocytic infiltration of lamina propria, crypt apoptosis (black arrows), and focal crypt destruction (white arrow). Scale bar = 100 µm.

Syngeneic grafts did not show any histological alterations at any of the two time points and revealed long, slender villi, continuous epithelium, and no apoptosis in the crypts. Three days after transplantation, the histology of the intestinal allografts was unremarkable, and did not show any signs of acute rejection or other abnormalities. However, at post-transplantation day six, all allografts showed swollen villus tips, significant lymphocytic infiltrate in the lamina propria, widespread crypt apoptosis, and focal crypt destruction, findings consistent with moderate rejection (Figure 1).

### 2.2. The Proteomic Analysis

**Figure 2:**
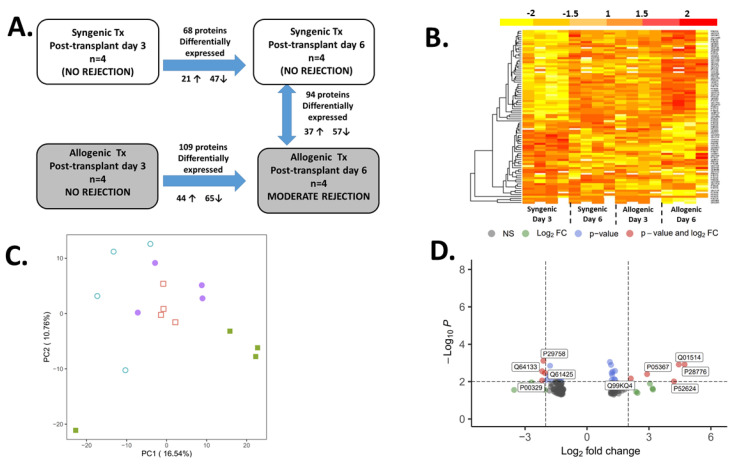
An outline of the proteomics analysis. (**A**) Summary of proteins differing between the two time points and groups; (**B**) Heat map showing the relative abundance and clustering of the 86 proteins identified across all four groups; (**C**) Principal component analysis (PCA) of the samples in the syngeneic group post-transplant day 3 (open circle) and day 6 (closed circle), and in the allogenic group at post-transplant day 3 (open square) and day 6 (closed square); (**D**) Volcano plot illustrating the fold change (log base 2) in protein expression in relation to the *p*-value (−log base 10) between non-rejecting (syngeneic) vs. rejecting (allogenic) grafts at day 6. Each dot represents a protein. Proteins at a significance level greater than 0.01 are in blue, those with a log2 fold change less than −2 and greater than 2 are in green, while proteins fulfilling both thresholds are in red, and their names are displayed.

The proteomic analysis identified 3172 proteins, 1522 of which were identified based on three or more unique peptides. Of these, 1087 proteins were detected in all samples. We identified 109 proteins that demonstrated a significant change (either increase or decrease) in the allogeneic grafts between day 3 and day 6. Ninety-four proteins were found differentially expressed between the rejecting (allogenic) grafts and the non-rejecting (syngeneic) grafts at day 6 after transplantation (Figure 2). Of these, the following eight proteins had the same alteration pattern as that found in syngeneic grafts (and were likely unrelated to AR) and were therefore excluded from the analysis: mitochondrial 3-ketoacyl-CoA thiolase (THIM), non-specific lipid-transfer protein (NLTP), Arginase 2 (ARGI2), cytoplasmic isocitrate dehydrogenase (IDHC), hydroxyacyl-coenzyme A dehydrogenase (HCDH), alcohol dehydrogenase 1 (ADH1), carbonyl reductase 1 (CBR1), and UDP-glucose 6-dehydrogenase (UGDH). The remaining 86 proteins had a changed pattern only found in the rejecting allografts. Eighteen proteins (21%) had a >2-fold change (either increase or decrease) while the other forty-six proteins (53%) revealed a 1.5–2-fold change compared with the levels before rejection. Interestingly, the expression of four proteins, serine protease inhibitor A3N (SPA3N), fibrinogen gamma (FIBG), annexin 13 (ANX13,) and NADPH-cytochrome P450 reductase (NCPR), increased in rejecting grafts at day 6 whereas they decreased in non-rejecting syngeneic grafts at the same time point compared with day 3.

In brief, the number of proteins that showed more than a 1.2-fold increased expression included enzymes (11), regulatory proteins, or transcription factors (16) and structural proteins (11). The identified number of proteins that decreased their expression by more than 20% during the rejection event included enzymes (28), regulatory proteins (9), and structural or functional proteins (10). Further details are presented in Table 1.

**Figure 3:**
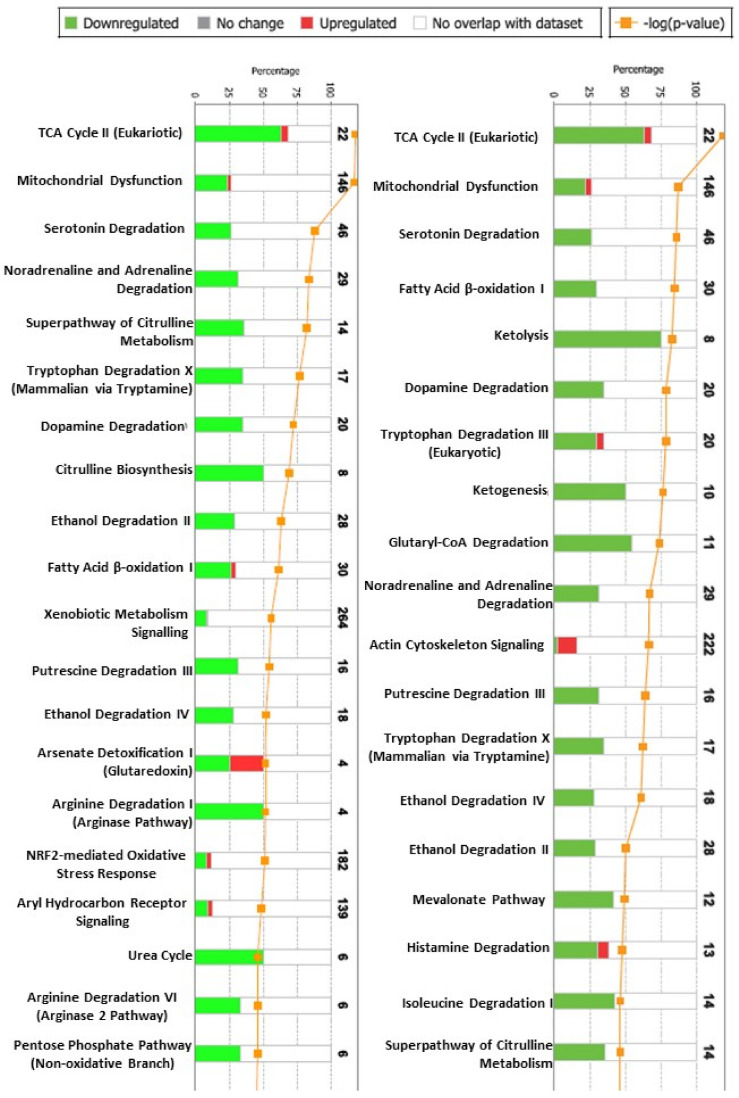
The alterations in the first 20 canonical pathways in allogenic grafts between post-transplant day 3 and day 6 (**left**) and between rejecting (allogeneic grafts) and non-rejecting (syngeneic grafts) at post-transplant day 6 (**right**) as revealed by the interactive pathway analysis. Downregulated pathways shown in green, upregulated pathways shown in red.

IPA interaction analysis found two hundred and ten canonical pathways differentially expressed between the syngenic (no rejection) and allogeneic grafts (moderate rejection) at day 6. In addition, two hundred and fourteen canonical pathways were found differentially expressed between the allogeneic grafts at day 3 (no rejection) and allogeneic grafts (moderate rejection) at day 6. The first 20 canonical pathways (ordered according to the magnitude of changes between the first and the second time point) in both allogenic and syngenic grafts are shown in Figure 3. Pathways involved in energy metabolism, TCA, substrate metabolism (glucose, fatty acid, amines and amino acid metabolism), and mitochondrial oxidative phosphorylation, all linked to oxidative stress response were among the most altered (mostly downregulated proteins).

The four biological networks that differed the most between allografts with or without rejection, as indicated by the IPA, were (i) cell-to-cell signaling and interaction, tissue development and cell cycle (35 proteins differing); (ii) energy production, lipid metabolism, small molecule biochemistry (33 proteins differing); (iii) cell death and survival, carbohydrate metabolism, lipid metabolism (21 proteins); and (iv) lipid metabolism, small molecule biochemistry, organismal functions (21 proteins).

### 2.3. Confirmatory Analysis: Western Blot Analysis and Immunohistochemistry

**Figure 4:**
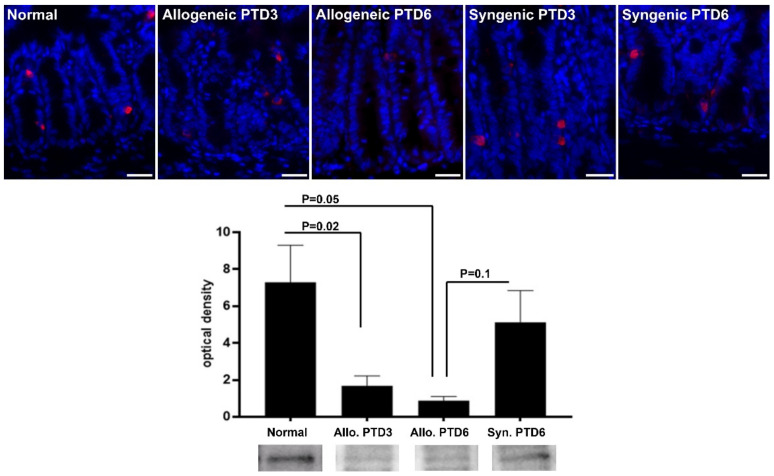
(**Upper panel**) Immunofluorescence microphotographs showing crypt cells positive for chromogranin A (red). Nuclei were stained blue using 40,6-diamidino-2-phenylindole. Original magnification 400×, scale bar 25 µm. (**Lower panel**) Western blot analysis for chromogranin A and representative immunoblot bands. The results from four separate experiments in each group are shown. PTD–post-transplant day.

To confirm the iTRAQ data from the pooled samples, we assessed the expression level of chromogranin A in individual samples using western blot analysis and immunofluorescence. This protein was selected as it was the second most downregulated protein as indicated by the proteomics analysis. In addition, chromogranin A is an analyte for which reliable, routine laboratory tests are already available. Immunoblotting for chromogranin A revealed downregulation in allogeneic, rejecting transplants on day 6 post-transplant when compared with the normal intestines (9.4-fold change, *p* = 0.05, Mann–Whitney U test) or syngeneic controls at the same time point (3.32-fold change, *p* = 0.1, Mann–Whitney U test; Figure 4).

Normal intestines had 1–2 cells positive for chromogranin A in each crypt (Figure 4). Syngeneic grafts had similar density and distribution of chromogranin-positive cells with that of normal intestines at both time points. At day 3 post-transplant, allografts had lower density and size of chromogranin A-positive cells whereas at during moderate rejection (post-transplant 6), most crypts were devoid of positive cells

## 3. Discussion

This study deciphers novel molecular mechanisms of intestinal allograft rejection in mice and represents the most comprehensive proteomic investigation on this topic to date. The analysis offers a simultaneous snapshot of several major processes and events during the acute rejection and maps its key players (e.g., showing the upregulation of specific proteins responsible for apoptosis and cell death, inflammation, cell migration, and antigen presentation). A few of these alterations such as impaired citrulline or tryptophan metabolism or increased calprotectin have been identified earlier in experimental and clinical studies [4,5], while several of our findings are novel for the intestinal transplantation setting.

The current results also revealed significant alterations in the tissue expression of several structural proteins. Due to the ongoing cell and tissue injury during rejection, this was an anticipated finding that we considered in the experimental design and explains why grafts with only a moderate rejection were assessed. Advanced AR would have involved significant tissue injury and mucosal loss, whereas during moderate AR, all mucosal compartments are preserved and the enterocyte mass is nearly intact. Hence, our experimental model allowed us to study a broad array of changes that occurred in a largely retained mucosa.

Besides changes in structural proteins, the analysis found quantitative changes of various enzymes and components of several cellular signaling pathways. Some of these pathways were identified as parts of the ongoing inflammation and tissue injury. As an example, we found a greatly increased expression of signal transducer and activator of transcription (STAT)-1, a transcription factor known to promote intestinal allograft rejection [17] or the upregulation of H-2 class I histocompatibility antigen, the murine correspondent of the human major histocompatibility complex class II. Furthermore, rejecting intestines showed increased expression of serum amyloid A, an acute-phase lipoprotein induced during inflammation or infection and has been shown to correlate with ICAM-1 or VCAM in patients with Crohn’s disease [18].

Several of the identified proteins are involved in maintaining the cellular redox status, modulate the oxidative stress, and provide cytoprotection against pro-oxidant stimuli. It is known that inflammation increases the level of reactive oxygen metabolites, resulting in oxidative stress due to an imbalance between antioxidants and reactive oxygen. Many of these classical antioxidant enzymes such as catalase, glutathione peroxidases, and peroxiredoxins directly inactivate reactive oxygen and nitrogen species, and have been suggested to be suitable biomarkers themselves for oxidative stress [19,20]. Other antioxidant enzymes such as 2,4-dienoyl-CoA reductase, 3-ketoacyl-CoA thiolase A, or glutathione-S-transferase recycle thiols or detoxify endogenous compounds such as peroxidized lipids and reactive secondary metabolites (such as aldehydes, peroxides, epoxides) [20]. The notable decrease in the tissue expression of several key antioxidant enzymes shown herein reflects a significant, ongoing oxidative stress and suggests that this is a mechanistic cause for cell injury related to intestinal acute rejection. This mirrors an earlier proteomics analysis that assessed rejection after rat liver transplantation. That study detected decreased tissue levels of catalase and aldehyde dehydrogenase and advocated that the imbalance in the reactive oxygen species scavenging machinery may have contributed to the dysfunction of hepatocytes and liver allografts [21]. Our results are also similar to a proteomics analysis of rejecting mouse hearts that found significantly decreased aconitate hydratase and increased pyruvate kinase isozyme M2 and glyceraldehyde-3-phosphate dehydrogenase (all related to the energy metabolism) [22]. Taken together, these findings suggest an impending energetic failure, in addition to the deteriorating antioxidant defense.

Our proteome analysis also revealed an obvious ongoing stress response in the rejecting grafts as indicated by the upregulation of several stress-related proteins, most notably heat shock protein (HSP) 90. Although acute rejection has been previously shown to induce various heat shock proteins in the intestine [10,23] and other organs [24], the significance of the heat shock response and its pathways remains unclear [25,26]. In addition, we found significant increases in the tissue expression of several central enzymes including uridine phosphorylase 1 (UPP1) and nicotinamide phosphoribosyltransferase (NAMPT), which are involved in key cellular functional mechanisms such as DNA repair and chromatin remodeling, secondary to the progressive, ongoing tissue injury (apoptosis, necrosis). Interestingly, the expression of the four proteins serine protease inhibitor A3N (SPA3N), fibrinogen gamma (FIBG), annexin 13 (ANX13), and NADPH-cytochrome P450 reductase (NCPR) increased in the rejecting grafts at day 6, while they decreased in non-rejecting syngeneic grafts at the same time point compared with day 3. Whereas the PCA analysis revealed a certain overlay of the non-rejecting groups, rejecting grafts did not overlap with any of the other datasets, indicating an alteration pattern rather specific to AR and giving hope to the search for new candidate biomarkers for intestinal rejection.

Chromogranin A, a neuroendocrine secretory protein produced by the enteroendocrine crypt cells, was selected for the confirmatory study as it was the second most downregulated protein in the rejecting intestines, as revealed by the proteomics analysis. Immunoblotting and immunofluorescence confirmed the decreasing trend in the rejecting allografts. This may be followed by lower levels in the blood or feces, suggesting a potential use of chromogranin A in blood or feces as a non-invasive rejection biomarker. Interestingly, the confirmatory analyses also indicated a trend toward lower chromogranin in syngeneic, non-rejecting grafts. The significance of this finding is unclear, although previous studies found a lower density of chromogranin A-positive cells in the mouse intestine following vagotomy [27] or in the prostate after its peripheral denervation [28]. Hence, lower chromogranin A expression may be, at least in part, secondary to graft denervation following transplantation.

The current analysis was restricted to proteins identified on the basis of three or more unique peptides. This arbitrary threshold made protein identification extremely accurate, but this selection may have omitted numerous other relevant proteins. Hence, an extended analysis of the remaining >200 proteins identified based on two unique peptides is mandated. A relative shortcoming of the present study is the use of homogenate from whole tissue samples. Thus, it is difficult to discriminate between the tissue compartments (i.e., mucosa, submucosa, muscular layer, vasculature, immune cells) where these changes have occurred. The results were obtained using intestinal transplantation in mice and without the use of immunosuppressive medication, and the intestine was transplanted heterotopically, without exposure to luminal alimentary stimuli or proximal trophic factors. However, despite the experimental setting, the clinical relevance of the current findings is apparent when considering the current findings of impaired citrulline metabolism during intestinal acute rejection [29] and the increased expression of S100-A8 (a constitutive part of calprotectin) [4]. As discussed earlier, citrulline has been shown to decrease following significant intestinal mucosal damage during severe rejection supposedly through the loss of enterocyte mass [5]. Our analysis identified altered expression of several enzymes central for citrulline biosynthesis. Hence, the current data suggest that, besides the loss of enterocytes, citrulline decrease may also be functional due to lower expression of upstream enzymes.

This study confirmed the proteomics finding for only one protein out of the 86 proteins, revealing an altered expression during intestinal AR. This investigation needs to be expanded to other proteins in the list, and analyzed with respect to corresponding alterations in the intestinal luminal content and in the blood, using both animal and human samples. In conclusion, this proteome-wide analysis indicated a significant ongoing oxidative stress during the acute rejection of the murine intestinal allograft and the exhaustion of several key redox mechanisms. Almost 100 proteins involved in numerous metabolic pathways altered their expression during allograft rejection. These pathways may include metabolites with potential new, non-invasive biomarkers for intestinal acute rejection.

## 4. Materials and Methods

### 4.1. Animals and Study Design

Male BalbC (donors) and C57BL6 (donors and recipients) mice weighing 25–30 g were used. Donor mice were fasted overnight before the explantation, while recipients had unrestricted access to food and water. The study closely followed the ethical regulations outlined by the NIH and the European Union and were reviewed and approved by the local committee of the Swedish Animal Welfare Agency (Dnr 287/99).

Heterotopic intestinal transplantation was performed in either allogeneic (BalbC donor to C57BL6 recipient) or syngeneic (C57BL6 to C57BL6) combinations. Previous experiments revealed that this fully allogeneic, high responder strain combination resulted in advanced rejection at post-transplant day six and severe rejection and animal death due to graft perforation and peritonitis by post-transplant day eight [16]. Mice (n = 4 per group and time point) were sacrificed at either day 3 and day 6 and graft segments were stored in formalin (histology) or snap-frozen (proteomics and western blot).

### 4.2. Surgery

Surgery was performed under 2% isoflurane anesthesia using a technique previously described [30]. In brief, the proximal half of the donor intestine was isolated by removing the duodenum, ileum, and colon and freeing the portal vein and the aorta above and below the emergence of the superior mesenteric artery. The intestinal graft was perfused in situ with cold, heparinized saline via the infrarenal aorta and stored in saline at 4 °C until recipient preparation (around 1 h). The graft was transplanted into the recipients using microvascular end-to-side anastomoses between the aortic patch containing the emergence of the superior mesenteric artery of the graft and the infrarenal recipient aorta. Venous drainage was achieved by anastomosing the portal vein of the graft and the recipient infrarenal vena cava, respectively, using 11/0 nylon sutures. The extrinsic, splanchnic nerves were not reconstructed. The proximal graft end was brought out as a stoma, while the distal end was anastomosed to the terminal ileum of the recipient. The recipient mice received a single intraperitoneal dose of cefuroxim (40 mg/kg) (Zinacef^®^, Glaxo Wellcome, UK) at the end of surgery. No immunosuppression was used.

### 4.3. Histology

Formalin-fixed intestinal graft segments were embedded in paraffin and cut into 5-micron sections. Hematoxylin-eosin slides were examined blindly by an experienced transplant pathologist and scored using a previously described scheme [30].

### 4.4. Immunofluorescence

Paraffin sections were deparaffinized and rehydrated, then antigen retrieval was performed by pressure cooking the slides in citrate buffer (pH 6) for 20 min. After species-specific blocking, slides were incubated overnight at 4 °C with antibodies against chromogranin A (PA5-77917, 1:250, Invitrogen AB, Lidingö, Sweden). Thereafter, slides were incubated with secondary antibody conjugated with Alexa 488 (1:500; Invitrogen). The sections were counterstained with 4′6′-diamidino-2-phenylindole, mounted with aqueous mounting medium (Vector Laboratories, Burlingame, CA, USA) and examined by fluorescence microscopy (Leica). Image acquisition and processing were performed using Leica LAS software.

### 4.5. Proteomic Analysis

#### 4.5.1. Sample Preparation

Samples were homogenized using a FastPrep-24 System (MP Biomedicals, Santa Ana, CA, USA). Samples were transferred to Lysis Matrix B tubes (MP Biomedicals, Santa Ana, CA, USA) containing 0.1 mm silica spheres. A total of 300 µL of lysis buffer (50 mM Triethylammoinium bicarbonate (TEAB) (Fluka, Sigma Alrdich, St Louis, MO), 8 M urea, 4% 3-[(3-cholamidopropyl)dimethylammonio]-1-propanesulfonate (CHAPS), 0.2% sodium dodecyl sulfate (SDS), 5 mM ethylenediaminetetraacetic acid (EDTA), adjusted to pH 8.5, was added to the tubes.

The protein concentration was determined using the Pierce 660 nm Protein Kit (Thermo Scientific, Basel, Switzerland) according to the manufacturer’s guidelines. For Tandem Mass Tag (TMT) labeling, 100 µg of the total protein of each sample and 100 µg of a pool containing equal amounts of all samples were diluted with two volumes of 0.5 M TEAB and 1 volume of 18 mΩH_2_O. One µL of 2% SDS was also added to each tube. The samples were reduced by the addition of 2 µL of 50 mM tris-(2-carboxyethyl) phosphine (TCEP) (Thermo Scientific, Basel, Switzerland), which was incubated at 37 °C for 1 h. The samples were subsequently alkylated with the addition of 1 µL of 200 mM methyl methanethiosulfonate (MMTS) (Fluka, Sigma Aldrich, St Louis, MO, USA) in gradient grade acetonitrile (Merck KGaA, Darmstadt, Germany) and incubated at room temperature for 10 min. The samples were digested with 4 µg of sequencing grade modified porcine trypsin (Promega, Madison, WI, USA) in 18 mΩH_2_O water to (Promega, Madison, WI, USA) overnight at 37 °C.

The digested samples were dried in a SpeedVac to ~25–30 µL and 70 µL of 0.5 M was then added to each tube as a preparation for labeling with the TMT^®^ (Thermo Scientific, Basel, Switzerland). TMT reagents were allowed to equilibrate to room temperature, and thereafter, 42 µL of gradient grade acetonitrile was added to each tube. The TMT reagent was transferred to the appropriate sample tube (see Table 1 for labeling details) and incubated at room temperature for 1 h. The reaction was quenched by the addition of 8 µL of 5% hydroxylamine (Thermo Scientific, Basel, Switzerland), which was incubated for 15 min at room temperature. After TMT labeling, the labeled samples were pooled and concentrated to ~50 µL in a SpeedVac in preparation for strong cation exchange (SCX) fractionation.

#### 4.5.2. Strong Cation Exchange Chromatography (SCX) of TMT Labeled Peptides

The concentrated peptides were acidified to below pH 3 by the addition of 25 µL 10% formic acid. The acidified labeled peptides were diluted with 25% gradient grade acetonitrile (Merck KGaA, Darmstadt, Germany) and injected onto a 2.1 mm i.d. × 10 cm length, 5 μm particle size, 300 Å pore size PolySULFOETHYL ATM strong cation-exchange (SCX) column (PolyLc Inc., Columbia, MD, USA) at a flowrate of 0.25 mL/min. SCX chromatography and fractionation was performed using an ÄKTA purifier system (GE Healthcare life science, Uppsala, Sweden) at 0.25 mL/min flow rate using the following gradient: 100% A (25 mM ammonium formate, pH 2.8 in 25% acetonitrile) for 10 min; 0–20% B (500 mM ammonium formate, pH 2.8 in 25% acetonitrile) for 20 min; 20–40% B for 10 min, and 40–100% B for 10 min and 100% B held for 10 min. UV absorbance at 280 nm was monitored while fractions were collected in tubes at 0.5 mL intervals and dried down in a Speedvac to approximately 50 µL. The 16 peptide containing fractions were desalted on PepClean C18 spin columns (Thermo Fisher Scientific, Inc., Waltham, MA, USA) according to the manufacturer’s guidelines.

#### 4.5.3. LC-MS/MS Analysis on LTQ-Orbitrap Velos

The desalted and dried fractions were reconstituted with 15 µL of 0.1% formic acid (Sigma Aldrich, St. Louis, MO, USA) in 3% acetonitrile and analyzed on a LTQ-Orbitrap Velos mass spectrometer (Thermo Fisher Scientific, Inc., Waltham, MA) interfaced with an in-house constructed nano-LC column. For each sample, a two micro-liter sample injection was made with an Easy-nLC autosampler (Thermo Fisher Scientific, Inc., Waltham, MA, USA), running at 200 nL/min. The peptides were trapped on a precolumn (45 × 0.075 mm i.d.) and separated on a reversed phase column, 190 × 0.075 mm, both packed in-house with 3 μm Reprosil-Pur C18-AQ particles (Dr. Maisch, Ammerbuch, Germany). The gradient was set to 0–70 min 5–35% acetonitrile in 0.2% formic acid, 70–80 min 35–80% acetonitrile in 0.2% formic acid, and the last 10 min at 80% acetonitrile in 0.2% formic acid.

Ions were injected into the LTQ-Orbitrap Velos mass spectrometer by electron spray ionization (ESI) under a spray voltage of 1.6 kV in positive ion mode with a capillary temperature of 250 °C. For MS scans, one microscan was performed at a 30,000 resolution (at m/z 400), and ions were detected within the mass range of m/z 400–1800. MS analysis was performed in a data-dependent mode. The top 10 most abundant doubly or multiply charged precursor ions, with a threshold count greater than 2000 and an isolation width (m/z) of 2.0 in each MS scan was selected for fragmentation (MSn) by stepped high energy collision dissociation (HCD). For MSn scans, one microscan was performed at a 7500 resolution (at m/z 400) with a mass range between m/z 120–2000 with stepped collision energies of 25%, 35%, and 45%, and a maximum injection time of 100 ms, and one repeat count was performed with a 30 s dynamic exclusion.

#### 4.5.4. Database Search and TMT Quantification

MS raw data files from all 16 SCX fractions for each TMT set were merged for relative quantification and identification using Proteome Discoverer^TM^ version 1.3 (Thermo Fisher Scientific, Inc., Waltham, MA, USA). A database search for each set was performed with the Mascot search engine (Matrix Science LTD., London, UK) using the Swissprot Database version 2.3 (Swiss Institute of Bioinformatics, Switzerland) with MS peptide tolerance of 10 ppm and MS/MS tolerance of 100 molecular mass units. Tryptic peptides with a maximum of one missed cleavage were accepted and variable modifications of methionine oxidation, cysteine methylthiolation, and fixed modifications of N-terminal TMT6plex and lysine TMT6plex were selected. Only spectra with a precursor mass between 400 and 8000 Da and a minimum peak count of 10 were chosen for identification.

The detected peptide threshold in the software was set to 1% false discovery rate by searching against a reversed database. Criteria used for positive protein identification were ≥3 peptides match, and an averaged ratio-fold change ≥1.2. For TMT quantification, the ratios of the TMT reporter ion intensities in MS/MS spectra ([M + H]+ m/z 126–131) from raw datasets were used to calculate fold changes between samples. Ratio was derived by Proteome Discoverer^TM^ using the following criteria: fragment ion tolerance as 80 ppm for the most confident centroid peak, TMT reagent purity correction factors were used, and missing quantification values were replaced with minimum intensity. To correct for experimental bias, all peptide rations were normalized by the median protein ratio, assuming a minimum count of 20 proteins had been observed. The co-isolation exclusion threshold of 30% was accepted for co-isolation interference. Only peptides unique for a given protein were considered for relative quantitation excluding those common to other isoforms or proteins of the same family. The quantification was normalized using the protein median. The results were then exported into MS Excel 2016 (Microsoft, Redmond, WA, USA) for manual data interpretation and statistical analysis.

#### 4.5.5. Bioinformatics Analysis of the Differentially Expressed Proteins

Pathway analysis (Ingenuity Pathway Analysis, IPA, Qiagen) was used to obtain further insight into potential cellular pathways that might be modified as a result of protein changes identified in present experiments. IPA automatically generated networks of gene, protein, small molecule, drug, and disease associations on the basis of “hand-curated” data held in a proprietary database. The identifiers (GI mouse identification number) of DEPs were uploaded as an Excel spreadsheet file onto the Ingenuity software (Ingenuity Systems, Redwood City, CA, USA). Each GI mouse identification number was mapped to its corresponding molecule in the Ingenuity Pathway Knowledge Base. The biological functions assigned to each network were ranked according to the significance of that biological function to the network. Networks of these proteins were algorithmically generated based on their connectivity and assigned a score. The score was used to rank networks according to how relevant they were to the proteins in the input dataset. The network identified was then presented as a graph indicating the molecular relationship between proteins. Finally, we compared the proteins differentially expressed between rejecting and non-rejecting grafts varying more than 20% between groups and time points

### 4.6. Western Blot Analyses of Intestinal Mucosa

Western blot protein analysis was performed using whole tissue frozen specimens as described earlier [31]. In brief, after electrophoresis and protein transfer on poly-vinyl-difluoride membranes, the membranes were blocked, then incubated overnight at 4 °C with primary antibody against chromogranin A (PA5-77917, 1:500, Invitrogen AB, Lidingö, Sweden). After repeated washings, a secondary antibody was applied for 1 h at room temperature and visualization was carried out using the chemiluminescent enzyme substrate CDP-Star (Tropix, Bedford, MA, USA). After repeated washings, a secondary antibody was applied for 1 h at room temperature and visualization was carried out using the chemiluminescent Clarity Western ECL (1705062, Bio-rad). The signal intensities of specific bands were detected and analyzed using a Chemi-doc Imaging system. Data were obtained via ImageLab software using Stain-free technology to perform total protein normalization. The Mann–Whitney U test was used for statistical analysis of western blot data; GraphPad Prism 6 (GraphPad Software, La Jolla, CA, USA) was used, and *p* values less than 0.05 were considered statistically significant.

## Figures and Tables

**Table 1:** List of proteins in the rejecting allografts with an altered protein expression relative to that found in syngeneic, non-rejecting grafts at the same time point (post-transplant day 6) as identified by iTRAQ-based quantitation.

Accession	Symbol	Description	Fold Change	Molecular Function	Biological Process
**Upregulated tissue expression**					
P28776	Ido1	Indoleamine 2,3-dioxygenase 1	4.72	Dioxygenase, Oxidoreductase	Inflammatory response
Q01514	Gbp1	Interferon-induced guanylate-binding protein 1	4.44	Hydrolase	Inflammatory response
P52624	Upp1	Uridine phosphorylase 1	4.21	Glycosyltransferase, Transferase	Inflammatory response
Q91WP6	Ser3na	Serine protease inhibitor	3.19	Protease inhibitor	Inflammatory response
P27005	S100a8	Protein S100-A8	3.17	Antimicrobial	Cell death and survival
P42225	Stat1	Signal transducer and activator of transcription 1	3.04	Activator, DNA-binding	Cell death and survival
P05367	Saa2	Serum amyloid A-2 protein	2.91	Cytokine	Inflammatory response
Q8VCM7	Fgg	Fibrinogen gamma chain	2.44	Binding protein	Hemostasis
O35744	Chi3l1	Chitinase-3-like protein 3	2.38	Antimicrobial	Inflammatory response
Q99KQ4	Nampt	Nicotinamide phosphoribosyltransferase	2.12	Cytokine, Glycosyltransferase	Cell death and survival
E9Q555	Rnf213	E3 ubiquitin-protein ligase RNF213	1.82	Hydrolase, Transferase	Angiogenesis
P01899	H2-d1	H-2 class I histocompatibility antigen, D-B alpha chain	1.69	Binding protein	Immunology
Q9R233	Tapbp	Tapasin	1.59	Binding protein	Immunology
Q9JIK5	Ddx21	Nucleolar RNA helicase 2	1.56	Binding protein	Immunology
P17918	Pcna	Proliferating cell nuclear antigen	1.55	DNA-Binding	Cell death and survival
P31001	Des	Desmin	1.52	Muscle protein	Cell structure
P26041	Msn	Moesin	1.49	Signal protein	Inflammatory response
Q60590	Orm1	Alpha-1-acid glycoprotein 1	1.46	Transport protein	Inflammatory response
P25206	Mcm3	DNA replication licensing factor MCM3	1.4	DNA-binding, Helicase, Hydrolase	Cell death and survival
P09405	Ncl	Nucleolin	1.36	Binding protein	Angiogenesis
P16858	Gapdh	Glyceraldehyde-3-phosphate dehydrogenase	1.35	Oxidoreductase, Transferase	Inflammatory response
P68033	Actc1	Actin, alpha cardiac muscle 1	1.35	Muscle protein	Cell movement
Q6NZJ6	Eif4g1	Eukaryotic translation initiation factor 4 gamma 1	1.35	Initiation factor, RNA-binding, Translational shunt	Cell death and survival
P52480	Pkm	Pyruvate kinase isozymes M1/M2	1.35	Allosteric enzyme, Kinase, Transferase	Cancer
Q9CPY7	Lap3	Cytosol aminopeptidase	1.32	Aminopeptidase, Hydrolase, Protease	Cell death and survival
Q9Z1Q5	Clic1	Chloride intracellular channel protein 1	1.32	Ion channel	Cellular growth and proliferation
Q99JG3	Anxa13	Annexin A13	1.31	Binding protein	Cell death and survival
P97372	Psme2	Proteasome activator complex subunit 2	1.29	Immunoproteasome assembly	Cell death and survival
Q61029	Tmpo	Lamina-associated polypeptide 2	1.27	DNA-Binding	Cell structure
P11499	Hsp90ab1	Heat shock protein HSP 90-beta	1.25	Chaperon	Cell death and survival
P05784	Krt18	Keratin, type I cytoskeletal 18	1.24	Structural protein	Cell structure
Q80 × 90	Flnb	Filamin-B	1.24	Actin-binding,	Cell movement
P60710	Actb	Actin, cytoplasmic 1	1.21	Muscle protein	Cell movement
P97371	Psme1	Proteasome activator complex subunit 1	1.21	Immunoproteasome assembly	Inflammatory response
P62137	Ppp1ca	Serine/threonine-protein phosphatase PP1-alpha catalytic subunit	1.2	Hydrolase, phosphatase	Cellular growth and proliferation
P37040	Por	NADPH--cytochrome P450 reductase	1.2	Oxidoreductase	Cellular function and maintenance
O08808	Diaph1	Protein diaphanous homolog 1	1.2	Actin-binding	Cell structure
**Downregulated tissue expression**					
Q9CZ13	Uqcrc1	Cytochrome b-c1 complex subunit 1, mitochondrial	0.79	Electron transport, Respiratory chain, Transport	Cellular function and maintenance
Q9ERG0	Lima1	LIM domain and actin-binding protein 1	0.79	Binding protein	Lipid metabolism
Q9D0F3	Aldh1b1	Protein ERGIC-53	0.79	Oxidoreductase	Cancer
Q921H8	Acaa1	3-ketoacyl-CoA thiolase A, peroxisomal	0.79	Acyltransferase, Transferase	Lipid metabolism
Q02819	Nucb1	Nucleobindin-1	0.78	DNA-binding, Guanine-nucleotide releasing factor	Cellular growth and proliferation
P09103	Pdia1	Protein disulfide-isomerase	0.78	Isomerases	Cell death and survival
Q9CY27	Tecr	Trans-2,3-enoyl-CoA reductase	0.78	Oxidoreductase	Lipid metabolism
Q9JII6	Ak1a1	Alcohol dehydrogenase	0.77	Dehydrogenase/reductase	Small molecule biochemistry
P28271	Aco1	Cytoplasmic aconitate hydratase	0.77	Lyase, RNA-binding	Cellular growth and proliferation
Q9JLQ0	Cd2ap	CD2-associated protein	0.76	Adapter protein	Cell cycle
Q99KI0	Acon	Aconitate hydratase, mitochondrial	0.76	Lyase	Cellular growth and proliferation
P47738	Aldh2	Aldehyde dehydrogenase, mitochondrial	0.75	Oxidoreductase	Small molecule biochemistry
P19783	Cox41	Cytochrome c oxidase subunit 4 isoform 1	0.75	Oxidoreductase	Cellular function and maintenance
P35700	Prdx1	Peroxiredoxin-1	0.74	Peroxidase	Cellular function and maintenance
P45952	Acadm	Medium-chain specific acyl-CoA dehydrogenase, mitochondrial	0.74	Oxidoreductase	Lipid metabolism
P24270	Cat	Catalase	0.74	Catalase	Cellular function and maintenance
Q60598	Cttn	Src substrate cortactin	0.72	Unknown	Cell structure
Q80XN0	Bdh1	D-beta-hydroxybutyrate dehydrogenase, mitochondrial	0.71	Dehydrogenase/reductase	Lipid metabolism
Q9EPB4	Pycard	Apoptosis-associated speck-like protein containing a CARD	0.71	Unknown	Inflammatory response
Q9DBS5	Klc4	Kinesin light chain 4	0.71	Motor protein	Cell movement
P99028	Uqcrh	Cytochrome b-c1 complex subunit 6, mitochondrial	0.71	Oxidoreductase	Cellular function and maintenance
Q9D855	Uqcrb	Cytochrome b-c1 complex subunit 7	0.71	Electron transport, Respiratory chain, Transport	Cellular function and maintenance
Q5SYD0	Myo1d	Myosin-Id	0.71	Motor protein	Cell structure
Q8K2B3	Sdha	Succinate dehydrogenase [ubiquinone] flavoprotein subunit, mitochondrial	0.7	Oxidoreductase	Cellular function and maintenance
Q9CQW5	Lgals2	Galectin-2	0.7	Binding protein	Unknown
Q99K01	Pdxdc1	Pyridoxal-dependent decarboxylase domain-containing protein 1	0.69	Decarboxylase, Lyase	Cell cycle
P10852	Slc3a2	4F2 cell-surface antigen heavy chain	0.68	transport protein	Cellular function and maintenance
Q3UMR5	Mcu	Coiled-coil domain-containing protein 109A	0.68	Calcium channel, Ion channel	Cellular function and maintenance
Q9Z2I8	Suclg2	Succinyl-CoA ligase subunit beta, mitochondrial	0.68	Ligase	Cellular function and maintenance
Q8VC30	Tkfc	Bifunctional ATP-dependent dihydroxyacetone kinase	0.66	Multifunctional enzyme	Cellular function and maintenance
Q9R100	Cdh17	Cadherin-17	0.65	Adhesion protein	Cell structure
P56391	Cox6b1	Cytochrome c oxidase subunit 6B1	0.65	Oxidoreductase	Cellular function and maintenance
P14152	Mdh1–2	Malate dehydrogenase, cytoplasmic	0.64	Oxidoreductase	Cellular function and maintenance
Q8C196	Cps1	Carbamoyl-phosphate synthase, mitochondrial	0.64	Ligase	Cellular function and maintenance
P31786	Acbp	Acyl-CoA-binding protein	0.63	Binding protein	Unknown
Q9DCN2	Nb5r3	NADH-cytochrome b5 reductase 3	0.61	Oxidoreductase	Lipid metabolism
P57016	Lad1	Ladinin-1	0.59	Anchoring filament	Cell structure
O09131	Gsto1	Glutathione S-transferase omega-1	0.58	Oxidoreductase, Transferase	Oxidative stress
Q8K0C9	Gmds	GDP-mannose 4,6 dehydratase	0.57	Lyase	Cellular function and maintenance
Q9CZS1	Al1b1	Aldehyde dehydrogenase X, mitochondrial	0.57	Oxidoreductase	Small molecule biochemistry
Q9CQ62	Decr	2,4-dienoyl-CoA reductase, mitochondrial	0.54	Oxidoreductase	Lipid metabolism
Q8R0Y6	Fthfd	10-formyltetrahydrofolate dehydrogenase	0.54	Oxidoreductase	Cellular function and maintenance
Q9D8W7	Ocad2	OCIA domain-containing protein 2	0.5	Unknown	Cancer
Q9QWG7	St1b1	Sulfotransferase family cytosolic 1B member 1	0.49	Sulfotransferase	Cellular function and maintenance
P29758	Oat	Ornithine aminotransferase, mitochondrial	0.47	Aminotransferase, Transferase	Cellular function and maintenance
Q64133	Aofa	Amine oxidase A	0.46	Oxidoreductase	Cellular function and maintenance
O88310	Itl1a	Intelectin-1a	0.37	Antimicrobial	Inflammatory response
P26339	Cmga	Chromogranin-A	0.31	Inhibitor protein	Immunology
P35230	Reg3b	Regenerating islet-derived protein 3-beta	0.28	Antibacterial protein	Immunology

## Data Availability

The data presented in this study are available on request from the corresponding author. The data are not publicly available due to further analysis ongoing.
